# DMAGCL: A dual-masked adaptive graph contrastive learning framework for predicting circRNA-drug sensitivity

**DOI:** 10.3389/fgene.2025.1721716

**Published:** 2025-11-26

**Authors:** Peng Wang, Yuqi Guo, Zejun Li, Di Tang, Mingming Qi, Zhanyi Zhu, Lichao Zhang

**Affiliations:** 1 School of Electronic Information, Hunan First Normal University, Changsha, Hunan, China; 2 School of Computer and Information Science, Hunan Institute of Technology, Hengyang, China; 3 School of Data Science and Artificial Intelligence, Wenzhou University of Technology, Wenzhou, China; 4 School of Software Quanzhou University of Information Engineering, Quanzhou, China; 5 School of Intelligent Manufacturing and Equipment, Shenzhen University of Information Technology, Shenzhen, China

**Keywords:** attention fusion classifier, circRNA, drug sensitivity prediction, dual-masked graph contrastive, multi-source feature integration

## Abstract

Circular RNAs (circRNAs) are a unique class of non-coding RNAs with stable covalently closed structures that play key regulatory roles in gene expression and drug response. However, experimental identification of circRNA-drug sensitivity remains labor-intensive. To overcome these limitations, we introduce DMAGCL, a Dual-Masked Graph Contrastive Learning framework, whose core innovations include: (1) a synergistic dual-masking strategy (path- and edge-level) that forces the model to learn robust representations against both macro-level path disruptions and micro-level edge noise; (2) an adaptive contrastive loss with a scheduled temperature parameter (t) to dynamically balance exploration and exploitation during training; and (3) an attention-based fusion classifier (AFC) that explicitly models complex cross-modal interactions between circRNA sequences and drug molecular graphs for adaptive multi-source information fusion. Comprehensive evaluations demonstrate that DMAGCL achieves state-of-the-art performance, attaining an average AUC of 0.8940 and AUPR of 0.9006 under five-fold cross-validation, and a slightly higher average AUC of 0.8982 under the more stringent ten-fold cross-validation, consistently surpassing strong baselines including GATECDA and MNGACDA. This performance advantage stems from our core design choices, as evidenced by systematic ablation studies confirming the indispensable and complementary roles of the dual-masking strategy and the effectiveness of the adaptive loss and fusion classifier. Case studies on four representative anticancer drugs (doxorubicin, gefitinib, sorafenib, and paclitaxel) achieved an average experimental validation rate of 80%, highlighting the framework’s predictive reliability and biological relevance. In conclusion, this study makes three primary contributions: (1) it introduces the novel DMAGCL framework, establishing a new paradigm for circRNA-drug association prediction via its synergistic dual-masking, adaptive learning, and attentive fusion components; (2) it delivers a highly robust and interpretable model with validated predictive reliability through extensive experiments and case studies (80% average validation rate); and (3) it provides a scalable computational tool that offers valuable insights for discovering novel circRNA-drug associations, understanding drug resistance mechanisms, and informing precision therapy design, with clear pathways for extension to other biological interaction tasks.

## 1 Introduction

Circular RNAs (circRNAs) exist stably through their covalently closed structure and have been proven to be key regulatory factors for tumor drug resistance (Chen and Yang, 2015; Suzuki and Tsukahara, 2014). As competitive endogenous RNAs, they can “sponge” miRNAs through miRNA response elements and thereby interfere with post-transcriptional silencing of mRNAs (Min et al., 2019). However, the complete molecular network of circRNA-drug sensitivity (CDS) remains to be elucidated. For instance, miRNAs can mediate post-transcriptional silencing by binding to the 3′-untranslated region of target mRNAs, while circRNAs can antagonize this process through molecular sponge effects (Bartel, 2004). circRNAs are pivotal regulators of tumor drug resistance (Min et al., 2019), yet computational elucidation of their interactions with drug sensitivity remains challenging (Bartel, 2004). Multiple studies have indicated that circHIPK3, Circ_0006528, etc. participate in tumor occurrence by regulating apoptosis and proliferation pathways (Hanahan and Weinberg, 2000), and can serve as diagnostic or prognostic markers (Li et al., 2018). At the level of drug resistance, circ_0076305 is upregulated by miR-186-5p to induce ABCC1 expression, thereby enabling non-small cell lung cancer to tolerate cisplatin (Liu et al., 2018); while circ_0072083 enhances the resistance of glioma to temozolomide through the miR-1252-5p/ALKBH5/NANOG axis (Wang et al., 2023). Although functional evidence is constantly emerging, experimental identification of CDS associations remains time-consuming and labor-intensive, and there is an urgent need for efficient computational frameworks to systematically analyze their network patterns. Additionally, in bladder cancer, CircNR3C1 can bind to BRD4 and interfere with the formation of the oncogenic complex between C-myc, thereby inhibiting tumor progression (Ding et al., 2021); studies have also shown that the ectopic expression of C-myc can partially reverse the tumor suppressive effect of this circRNA *in vivo* (Xie et al., 2020). These findings systematically reveal the important regulatory functions of circRNAs in tumor resistance. However, the complete molecular network of their interaction with drug sensitivity still needs to be further elucidatedExtensive experiments demonstrate that DMCL achieves state-of-the-art performance. Case studies on four anti-cancer drugs show an average experimental validation rate of 80%, underscoring its predictive reliability and biological relevance. This work provides a powerful computational tool and offers new insights into non-coding RNA mechanisms in drug resistance.

In the circRNA and drug sensitivity studies, the statistical models based on correlations play a crucial role. The core of these models lies in using computational methods to predict and uncover potential circRNA-drug sensitivity correlations, thereby effectively avoiding the time-consuming and labor-intensive drawbacks of traditional biological experiments. Computational models offer an efficient approach for large-scale identification of such associations. Existing methods primarily rely on strategies such as graph neural networks (GNNs), multi-source feature integration, or random walks. Multi-source biological information integration and machine learning algorithms have become the mainstream approach for predicting drug sensitivity of circRNAs(Wei et al., 2023a). By introducing graph regularization into protein language models, it has been verified that graph constraints can improve the prediction of TCR-epitope binding specificity (Fu et al., 2025b); Zhou et al. proposed a global-local dual perspective to achieve multi-scale feature fusion of drug-protein interactions (Zhou et al., 2024b). The multi-source feature framework emphasizes the crucial regulatory role of circular RNAs in drug sensitivity and efficacy (Yin et al., 2024), while MNCLCDA reveals the association between circular RNAs and drug resistance and tumor progression through mixed neighborhood contrast learning (Li et al., 2023). Given the natural advantages of graph neural networks in complex biological networks, studies usually combine attention mechanisms to strengthen key features (Ru et al., 2022): MNGACDA uses graph autoencoders to construct multimodal networks (Yang and Chen, 2023), DeepWalk-GAT combines convolutional neural networks to extract topological sequence information (Li et al., 2023), Graph Attention Autoencoder (Deng et al., 2022), HETACDA (Xiao et al., 2023), Double-Layer Multi-Core Attention Network (Lu et al., 2023), and multimodal graph representation framework (Liu Z. et al., 2025) all effectively predict circular RNA-drug sensitivity through computational means, compensating for the lack of experimental throughput (Ru et al., 2021a). However, these methods have inherent limitations: GNN models are susceptible to information redundancy and noise within networks, and their decision-making process often lacks transparency. Similarity-based methods, on the other hand, struggle to capture complex non-linear biological relationships. To address these challenges, we propose a double-mask contrastive learning framework. The core innovation involves the simultaneous application of a path mask and an edge mask. The path mask is designed to deconstruct high-order semantic information in heterogeneous circRNA-drug relationships, while the edge mask enhances the model’s robustness to local critical interactions. This dual design effectively suppresses information redundancy and noise in biological networks through contrastive learning. Furthermore, we introduce a dynamic temperature parameter strategy that adaptively optimizes the learning process based on the training state. Random walks and similarity measures are intertwined, forming a network that, through topological propagation, triggers potential correlations: an asymmetric dual-walk strategy re-evaluates the connection between circular RNAs and diseases, providing algorithmic support for rapid target identification (Toprak, 2024). The multi-head attention mechanism is used to convert the potential vocabulary between non-coding RNAs and proteins into interaction codes (Zhou et al., 2023). Graph collaborative filtering and multi-view contrastive learning are combined, enabling miRNAs to confront their own sensitivity to drugs in the generated embedding space (Wei et al., 2023b). Pairwise learning guides dual graph convolution to shape a clearer interface representation of ncRNA-protein pairs (Zhuo et al., 2022), while the head-tail sampling protocol extracts key nodes from the sparse interaction wasteland, providing replenishment for downstream GNNs(Wei et al., 2023a). Benchmarking and framework parallel development: an evaluation standard for circular RNA-disease prediction was established and quietly expanded to the field of drug sensitivity (Lan et al., 2023); through the method of extracting graph skeletons and fusing attention, researchers gradually revealed the progressive process of non-coding RNA-mediated drug resistance (Zhang et al., 2023); a comprehensive study revealed how deep learners locate binding sites of RNA-binding proteins on circular RNAs and how this binding implicitly regulates drug responses (Wang Z. et al., 2025). Multidimensional data convergence, complex algorithms take off, the statistical-graph hybrid now charts a high-throughput, low-cost route for drug sensitivity based on circular RNAs, accelerating the transformation of potential biomarkers and therapeutic targets from the realm of stars to clinical reality (Ru et al., 2021b).

Network models have emerged as a pivotal tool in bioinformatics and systems biology for modeling complex biological processes and diseases. For instance, Peng et al. developed an explainable multi-scale framework for circRNA-miRNA interaction prediction, highlighting the utility of multi-scale feature engineering in circRNA research (Peng et al., 2025b). In another study, Peng et al. introduced metaCDA, a meta-learning framework for circRNA-driven drug discovery, showcasing the potential of adaptive aggregation and meta-knowledge in circRNA studies (Peng et al., 2025a). By modeling biological entities and their interactions as graphs, network science has been widely applied in protein-protein interaction networks, gene regulatory networks, and brain connectomes. For instance, Milan and Canataro applied network inference to the research of cancer and neurodegenerative diseases (Milano and Cannataro, 2023); through the heterogeneous GCN combined with pseudo-path bidirectional attention (Niu et al., 2025), NSL2CD’s adaptive subspace embedding (Xiao et al., 2021b), AAECDA’s MSCNN-adversarial autoencoder pipeline (Wang Y. et al., 2025), and Xiao’s propagation, path, matrix and depth-based classification framework (Xiao et al., 2021a), as well as the cross-modal ternary attention in ET-PROTACs (Cai et al., 2025), Kolmogorov-Arnold drug KANs (Fu et al., 2025a), and masked path GAM-MDR (Zhou et al., 2024a), they jointly advanced the frontier of topological research. These studies have jointly promoted the development of circRNA-drug sensitivity association prediction based on network topological structures, providing new perspectives and methods for biomarker identification and drug development in complex diseases.

Machine learning is playing an increasingly important role in drug discovery and prediction, especially in drug sensitivity prediction. In the field of predicting drug sensitivity for cancer cell lines, even when the training samples are limited, traditional molecular fingerprints have been surpassed or even equalled by end-to-end TextCNN (Baptista et al., 2022). Breakthroughs have also been frequently observed in the circRNA domain, such as LSNSCDA, which overcomes the limitations of fixed step size and negative sample noise through a local smoothing graph neural network and reliable negative sampling (Fan et al., 2024); MAGSDMF framework, which precisely captures the potential circRNA-drug sensitivity associations through multiple attention, graph learning, and deep matrix decomposition (Ai et al., 2024); DCDA uses a feedforward-self-encoding hybrid to integrate multi-source data to shape circRNA-disease features and performs exceptionally well (Turgut et al., 2023); SNFTPGd-CDA achieves high AUC in nonlinear fusion of multi-source information through similar network fusion-tensor product graph diffusion parallel cascaded forest (Liu et al., 2024); JLCRB multi-view collaborative representation network enhances cross-view consistency to locate circRNA binding sites, and the average AUC has reached a new high (Du and Xue, 2022). Looking at the entire process of drug development, ModDRDSP uses deep bidirectional GRU and message passing networks to jointly depict multimodal drug information, and then integrates cell line multi-omics data, and is completed by a deep forest for sensitivity prediction, with the model’s performance comprehensively leading existing methods (Song et al., 2024).

In the research on the association between circRNAs and drug sensitivity prediction, statistical models based on correlations, models based on graph neural networks (GNN) and attention mechanisms, as well as models based on random walks and similarity measures each have their own advantages and limitations. The statistical model based on 155-dimensional correlation coefficients integrates multi-source biological data and employs machine learning algorithms to construct a prediction framework. It can simultaneously capture correlation signals in multiple dimensions such as expression profiles, drug structures, and clinical phenotypes, thereby enhancing prediction coverage and accuracy (Chen et al., 2022). Due to its high dependence on data quality and completeness, this method is prone to overfitting when the training samples are insufficient or the feature dimensions are excessive, resulting in a decline in generalization performance. In contrast, the combination of graph neural networks and attention mechanisms utilizes topological structure information for embedding learning of circRNAs and drugs, highlighting key node features through dynamic weights, demonstrating strong association mining capabilities in large-scale network scenarios (Li X. et al., 2025). However, this strategy has high computational costs, high memory usage, and a large number of hyperparameters, resulting in significant tuning costs. With the random walk and similarity measurement methods constructing a similarity network of biological entities and performing information propagation on it, potential associations can be rapidly scanned with linear complexity, suitable for initial screening tasks of large datasets (Li M. et al., 2025). Its performance is highly dependent on the accuracy of similarity measurement, and because the model is essentially a linear method, it is difficult to depict the nonlinear interactions within the biological system, resulting in insufficient performance in complex association prediction scenarios. Moreover, during the process of dimensionality reduction or network embedding, important biological details may be lost, affecting the precision of the prediction.

In summary, these three types of methods have distinct characteristics and complement each other in the prediction of the association between circRNA and drug sensitivity. Future research can integrate the advantages of these methods, such as combining the interpretability enhancement technology of GNN and the integration of multi-source features, to improve the prediction accuracy and biological relevance (Ma et al., 2024). At the same time, introducing data augmentation techniques, interpretability tools, and establishing standardized benchmark test datasets and evaluation indicators will help further optimize the model performance and promote the progress of this field (Liu T. et al., 2025). Moreover, exploring the application of these methods in other biomedical fields, such as gene regulatory networks and protein interaction networks, will further verify their universality and effectiveness.

## 2 Methods

**FIGURE 1:**
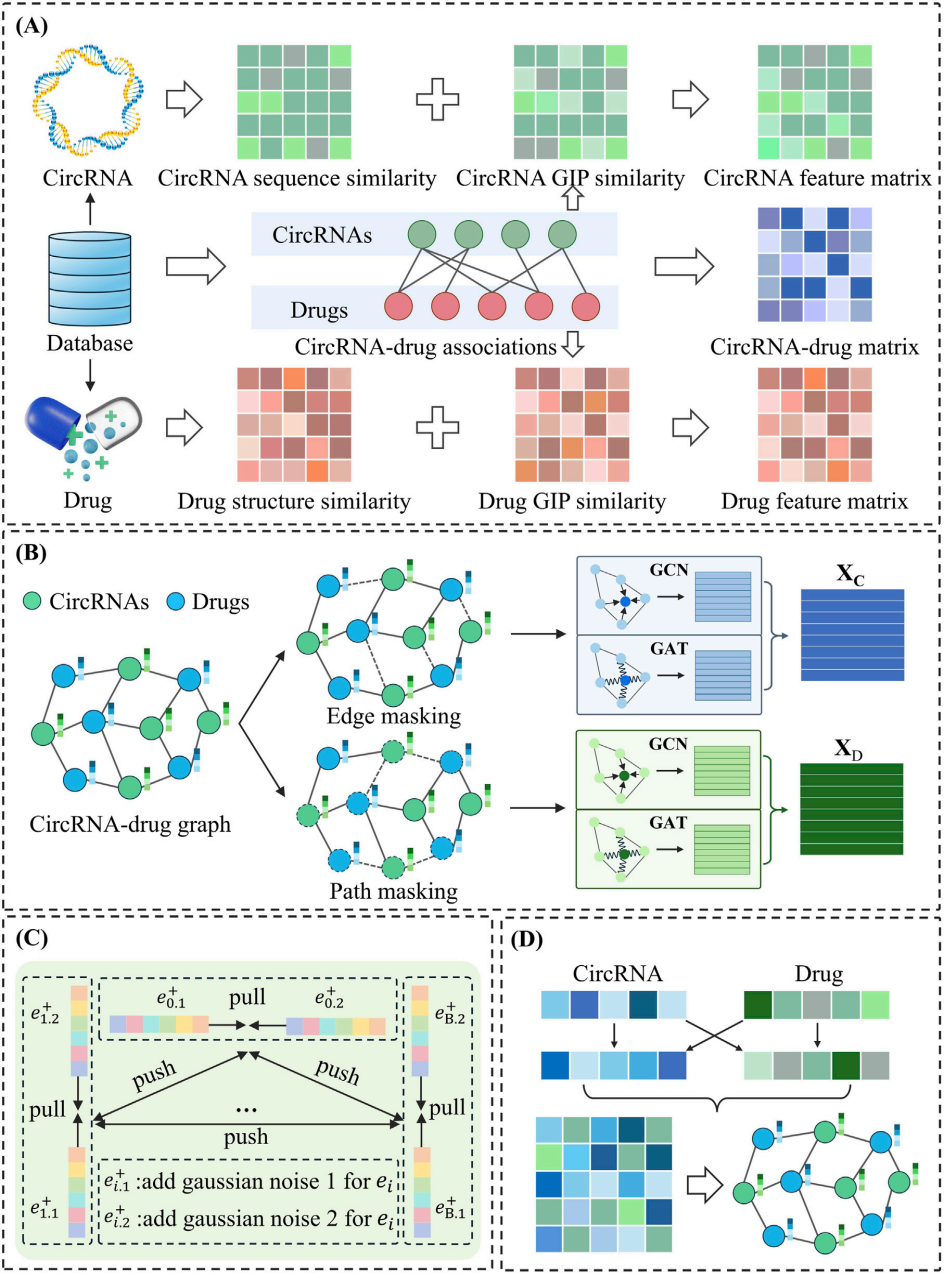
The computational flowchart of DMAGCL. **(A)** CircRNA and drug feature extraction **(B)** Dual view masking graph learning, **(C)** Adaptive contrastive learning **(D)** circRNA-drug graph resonstration.

We put forward a novel framework entitled DMAGCL. This framework is specifically designed to concurrently tackle three crucial challenges during the process of graph representation learning: information redundancy, negative sample noise, and class imbalance issues. Through an innovative architectural design, our method integrates graph encoding, masked reconstruction, dynamic negative sampling, ensemble learning, and ranking optimization into a unified end-to-end learning objective. This integration allows for the coordinated evolution of pseudo-label generation, sampling distribution adjustment, model ensemble, and evaluation metric optimization throughout the training process. The overall process is shown in Figure 1.

### 2.1 Multi-source feature extraction

To comprehensively characterize the characteristics of nodes in the network, we systematically extracted multi-source features from two dimensions: circRNAs and drugs. We then constructed a unified feature representation through an effective fusion strategy, providing high-quality feature inputs for subsequent graph representation learning. For circRNA nodes, we integrated two complementary feature sources. Based on the nucleic acid sequence information of circular RNA host genes, we used the edit distance algorithm to calculate the similarity between sequences, and constructed a circRNA sequence similarity matrix $R_{\text{seq}}\in R^{M\times M}$ . The elements of this matrix are defined as:

$$R_{\text{seq}}\left(i,j\right)=1-\frac{D_{\text{edit}}\left({Seq}_{i},{Seq}_{j}\right)}{\max\left(\left|{Seq}_{i}|,|{Seq}_{j}\right|\right)},$$ (1)

Here, $D_{\text{edit}}$ represents the minimum number of editing operations required to transform one sequence into another, and ${\text{Seq}}_{i}$ denotes the host gene sequence of the i -th circRNA. Additionally, based on the known circRNA-drug sensitivity correlation matrix $A\in R^{M\times N}$ , we constructed the Gaussian interaction spectral kernel similarity matrix $R_{\text{gip}}\in R^{M\times M}$ .

$$R_{\text{gip}}\left(i,j\right)=\exp\left(-\eta_{c}{\left\|A\left(i,:\right)-A\left(j,:\right)\right\|}^{2}\right),$$ (2)

The nuclear bandwidth parameter $\eta_{c}$ is determined by the following formula.

$$\eta_{c}={\left(\frac{1}{M}\overset{M}{\underset{k=1}{\sum }}{\left\|A\left(k,:\right)\right\|}^{2}\right)}^{-1}.$$ (3)

Ultimately, we obtained the comprehensive feature matrix $X_{c}\in R^{M\times M}$ of the circular RNA through conditional weighted fusion: when sequence similarity is available, the average value of the two similarities is taken; otherwise, only the Gaussian interaction spectral similarity is retained. In terms of the construction of drug characteristics, we adopted a similar dual-source feature fusion strategy. Based on the chemical structure information of the drugs, we used topological fingerprints and Tanimoto coefficients to calculate the structural similarity between drugs, forming a drug structure similarity matrix $D_{\text{stru}}\in R^{N\times N}$ .

$$D_{\text{stru}}\left(i,j\right)=\frac{\left|F\left(d_{i}\right)\cap F\left(d_{j}\right)\right|}{\left|F\left(d_{i}\right)\cup F\left(d_{j}\right)\right|},$$ (4)

Here, $F\left(d_{i}\right)$ represents the topological fingerprint feature vector of drug $d_{i}$ . Meanwhile, based on the same circRNA-drug sensitivity association matrix A , we constructed the Gaussian interaction spectral kernel similarity matrix $D_{\text{gip}}\in R^{N\times N}$ ,

$$D_{\text{gip}}\left(i,j\right)=\exp\left(-\eta_{d}{\left\|A\left(:,i\right)-A\left(:,j\right)\right\|}^{2}\right)$$ (5)

The parameter $\eta_{d}$ is determined by the following formula:

$$\eta_{d}=\frac{1}{N}\overset{N}{\underset{k=1}{\sum }}{\left\|A\left(:,k\right)\right\|}^{2}$$ (6)

The similarity features of the two drugs are fused through the same weighted strategy under the same conditions, resulting in a comprehensive feature matrix $X_{d}\in R^{N\times N}$ for the drugs. Based on the above feature matrix and the known correlation between circRNAs and drug sensitivity, we constructed a heterogeneous network H=(V,E) , where the node set V=C∪D includes all circRNA and drug nodes, and the edge set E integrates three types of connection relationships: circRNA - circRNA similarity edges, drug - drug similarity edges, and circRNA - drug association edges.

### 2.2 Dual masked graph views

While graph structure is vital for representation learning, standard GNNs can be limited in capturing complex topological dependencies. To address this, we propose a self-supervised contrastive learning framework with a dual-masking strategy to enhance the robustness and quality of graph representations. This method constructs two masking views with different structural perturbation intensities, capturing local structural patterns at the path level and edge level respectively, and leveraging the contrastive learning mechanism to improve the model’s perception of graph structure information.

Specifically, we designed two complementary masking modules: the Path Masking Module (MaskPath) and the Edge Masking Module (MaskEdge). The Path Masking Module randomly samples the node paths in the graph and performs overall masking on the nodes and their associated edges within the path, thereby generating the first perturbed view $G_{1}$ . This operation can simulate the absence of local connectivity patterns in the graph, forcing the model to still infer the dependencies between nodes even when the paths are incomplete. The Edge Masking Module is more granular, randomly selecting some edges in the graph for masking, generating the second perturbed view $G_{2}$ . This operation aims to weaken the local direct connections, enabling the model to pay more attention to other structural cues within the neighborhood. We introduce two complementary masking modules: MaskPath and MaskEdge. MaskPath samples and masks entire node paths, creating view $G_{1}$ and compelling the model to recover missing connectivity patterns. MaskEdge operates at a finer granularity by randomly dropping individual edges to form view $G_{2}$ , forcing the model to rely on broader neighborhood cues. This dual approach ensures that the model learns robust representations against both macro-level path disruptions and micro-level edge noise. The two masking operations can be formally represented as:

$$G_{1}=\text{MaskPath}\left(G,\alpha \right)$$ (7)

$$G_{2}=\text{MaskEdge}\left(G,\beta \right)$$ (8)

Among them, α and β are the intensity control parameters for the path mask and edge mask, respectively. They are used to adjust the range and degree of the masks, thereby controlling the disturbance intensity and diversity of the view.

We employ a shared-weight graph encoder E to map both masked views to a unified representation space. The encoder processes each view independently, generating corresponding graph-level representations $Z_{1}$ ) and $Z_{2}$ :

$$Z_{1}=E\left(G_{1}\right)$$ (9)

$$Z_{2}=E\left(G_{2}\right)$$ (10)

The encoder E can be implemented by combining a graph convolutional network (GCN) and a graph attention network (GAT). After obtaining the view representations, we use the adaptive contrastive loss as the contrastive target. By maximizing the mutual information between the representations of the same graph under different masking strategies, the model is guided to learn representations that are robust to structural perturbations. During the model training process, the input graph G is first subjected to path masking and edge masking to generate two complementary perturbed views $G_{1}$ and $G_{2}$ . Subsequently, the shared graph encoder extracts the graph-level representations $Z_{1}$ and $Z_{2}$ of the two. Next, the similarity of the representations is calculated, and a contrastive learning task is constructed based on the loss. Finally, the encoder parameters are optimized through the backpropagation mechanism to minimize the contrastive loss. This training strategy enables the model to extract common structural information from different masking perspectives, thereby effectively enhancing the modeling ability of local neighborhood dependencies and improving the quality and generalization of graph structure representations.

### 2.3 Multi-view graph encoder

In the field of graph neural networks, the effective modeling and feature extraction of multi-view graph data has always been a key and challenging research direction. Since multi-view graph data typically contains heterogeneous structural information from different sources or different feature spaces, how to fully integrate the effective features from each view and retain their inherent topological relationships is an important issue for improving the representational learning ability of graphs. To effectively integrate heterogeneous information from our constructed multi-view graph (e.g., sequence similarity, GIP similarity), we propose a Multi-View Graph Encoder. This encoder combines GCN for intra-view feature extraction and GAT for inter-view fusion, ensuring a balanced and comprehensive representation. The core design of this method consists of two main stages: local structure modeling within views and global feature fusion between views. Firstly, for the structural characteristics within each view, GCN is used to aggregate the neighborhood of nodes to capture the specific local topological patterns of the view. Specifically, for the v -th view, the node feature matrix is $X^{\left(v\right)}$ and the adjacency matrix is $A^{\left(v\right)}$ . To enhance training stability and retain the node’s own features, self-loops are introduced to construct an augmented adjacency matrix ${\tilde{A}}^{\left(v\right)}=A^{\left(v\right)}+I$ , and the corresponding degree matrix $\tilde{D}$ is calculated. The node feature update formula in the GCN layer is:

$$H^{\left(v\right)}=\sigma \left({\tilde{D}}^{-1/2}{\tilde{A}}^{\left(v\right)}{\tilde{D}}^{-1/2}X^{\left(v\right)}W^{\left(v\right)}\right),$$ (11)

Here, $W^{\left(v\right)}$ represents the trainable weight matrix specific to the view, and σ is the nonlinear activation function. Through this step, the smooth propagation of node features within each view and the structured-aware representation learning can be achieved.

After obtaining the node representations of each view, in order to further integrate the information from multiple views, this paper introduces the GAT mechanism to adaptively learn the contribution weights of different views to the current view. Specifically, for the target view v , its output representation after integrating the features of other views is:

$$H_{\text{attn}}^{\left(v\right)}=\sigma \left(\overset{V}{\underset{u=1}{\sum }}\alpha_{vu}H^{\left(u\right)}\right)$$ (12)

Here, $\alpha_{vu}$ represents the attention weight from view u to view v , reflecting the significance of view u in supplementing the features of view v . The calculation of this weight employs the following attention mechanism:

$$\alpha_{vu}=\frac{\exp\left(\text{LeakyReLU}\left(a^{T}\left[H^{\left(v\right)}\|H^{\left(u\right)}\right]\right)\right)}{\sum_{u=1}^{V}\exp\left(\text{LeakyReLU}\left(a^{T}\left[H^{\left(v\right)}\|H^{\left(u\right)}\right]\right)\right)}$$ (13)

Here, a represents a learnable attention parameter vector, and the symbol ‖ denotes the feature concatenation operation. Through this mechanism, the model can automatically identify the complementarity among different views and endow the information fusion process with greater interpretability.

Finally, the feature representations of each view, weighted by attention, are integrated and input into a fully connected layer or a specific downstream task module to generate the final graph representation or node representation. The integrated output can be expressed as:

$$Z=\sigma \left(W\left[H_{\text{attn}}^{\left(1\right)},H_{\text{attn}}^{\left(2\right)},\ldots ,H_{\text{attn}}^{\left(V\right)}\right]\right)$$ (14)

Here, W represents the globally trainable weight matrix, and σ is the activation function of the output layer. This integration strategy can integrate the advantages of each view, enhancing the model’s ability to model complex graph structures and multi-source information.

In conclusion, the multi-view graph encoder proposed in this paper combines the advantages of GCN in modeling the internal structure of views with the flexibility of GAT in integrating views, which not only effectively extracts the local features of each view, but also captures the global correlations between views. Thus, it achieves more comprehensive and robust feature learning in multi-view graph data processing.

### 2.4 Adaptive contrastive loss

As a robust and effective alternative, we introduce a Scheduled Contrastive Loss featuring a dynamic temperature parameter (t) that follows a predefined linear decay schedule. Specifically, this mechanism sets a higher temperature value in the early training stage to encourage the model to conduct more extensive exploration and avoid prematurely settling into suboptimal solutions; while gradually reducing the temperature value in the later training stage to enhance the model’s ability to distinguish between positive and negative samples, thereby improving its generalization performance.

The mathematical definition of the adaptive contrastive loss function is as follows. Let the input feature vectors be $z_{i}$ and $z_{j}$ , where i and j represent the positive sample pairs, and the other samples k (where k≠i,j ) are regarded as negative samples. The loss function can be expressed as:

$$L_{\text{adaptive}}=-\log\frac{\exp\left(\frac{z_{i}\cdot z_{j}}{\tau \left(t\right)}\right)}{\sum_{k=1}^{K}\exp\left(\frac{z_{i}\cdot z_{k}}{\tau \left(t\right)}\right)}$$ (15)

Here, $z_{i}\cdot z_{j}$ represents the dot product similarity between the feature vectors, K is the total number of negative samples, and τ(t) is a dynamic temperature parameter that adjusts according to the change in the training iteration number t . The definition of the dynamic temperature parameter τ(t) is:

$$\tau \left(t\right)=\tau_{\text{max}}-\frac{t}{T}\left(\tau_{\text{max}}-\tau_{\text{min}}\right)$$ (16)

Here, $\tau_{\text{max}}$ (initial temperature) encourages broad exploration early in training by smoothing the loss landscape. $\tau_{\text{min}}$ (final temperature) sharpens the focus on hard negatives later for better discrimination. T is the total number of iterations, governing the cooling schedule. This linearly decreasing temperature strategy simulates an ‘annealing’ process. It provides a structured learning curriculum for the model: starting with robust, noise-tolerant learning and progressively transitioning to high-precision discrimination. In the initial stage of training, a higher temperature enables the smooth handling of the similarity difference between positive and negative samples, thereby reducing the model’s sensitivity to noise and encouraging more diverse feature learning; as training progresses, the temperature gradually decreases, and the model gradually focuses on precisely distinguishing positive and negative samples, which helps to improve the discriminative power of features.

During the training process, we first initialize the key parameters, including $\tau_{\text{max}}$ , $\tau_{\text{min}}$ and the total number of iterations T . These parameters can be adjusted according to the data distribution of the specific task and the complexity of the model, for example, through cross-validation to optimize the selection. In each training iteration t , the temperature parameter τ(t) is updated in real time using the formula $\tau \left(t\right)=\tau_{\text{max}}-\frac{t}{T}\left(\tau_{\text{max}}-\tau_{\text{min}}\right)$ . Then, for each positive sample pair $\left(z_{i},z_{j}\right)$ , the similarity matrix between it and all other samples (including positive and negative samples) is calculated, and the contrastive loss $L_{\text{adaptive}}$ is computed using the current dynamic temperature τ(t) . The model parameters are updated using the backpropagation algorithm to minimize the loss function, while monitoring the training loss and validation set performance to avoid overfitting. This dynamic temperature adjustment mechanism not only effectively alleviates the problems of premature convergence and overfitting caused by fixed temperature parameters, but also adaptively balances the exploration and exploitation phases of the model, thereby improving the training efficiency while enhancing the generalization ability of the model on unseen data. Moreover, the flexibility of this method makes it easy to integrate into various contrastive learning frameworks, providing a scalable foundation for subsequent research.

### 2.5 Attention fusion classifier

Simple feature fusion (e.g., concatenation) fails to capture complex cross-modal interactions between circRNA sequences and drug molecular graphs. We therefore propose an Attention-based Fusion Classifier (AFC) to dynamically model these correlations and adaptively fuse multi-source information. Compared with traditional feature concatenation methods, AFC incorporates a multi-head attention structure, which can extract and fuse the interaction information between the circRNA sequence mode and the drug molecule graph mode from multiple subspaces, effectively enhancing the discriminative ability of the feature representation. Additionally, to further improve the robustness and generalization ability of the model, the classification layer adopts a hierarchical multi-layer perceptron (MLP) structure and combines batch normalization (BatchNorm) for regularization processing. Experiments have shown that AFC exhibits superior performance in multiple multimodal classification tasks.

Specifically, the core component of AFC is the multi-head attention mechanism. This mechanism can dynamically adjust the fusion weights based on the correlation between the input features of the circRNA sequence mode and the drug molecule graph mode, thereby focusing on the cross-modal information that is more helpful for the current task. Assuming the input is the feature embeddings of the circRNA sequence mode and the drug molecule graph mode, which are $F_{\text{circ}}\in R^{n\times d_{\text{circ}}}$ and $F_{\text{drug}}\in R^{n\times d_{\text{drug}}}$ , where n is the number of samples and $d_{\text{circ}}$ and $d_{\text{drug}}$ are the feature dimensions of the corresponding modes. First, the features of the circRNA sequence mode and the drug molecule graph mode are linearly transformed to a unified latent space dimension d :

$$U=F_{\text{circ}}P_{U}\in R^{n\times d},\;S=F_{\text{drug}}P_{S}\in R^{n\times d},\;T=F_{\text{drug}}P_{T}\in R^{n\times d}$$ (17)

Among them, $P_{U}\in R^{d_{\text{circ}}\times d}$ , $P_{S}\in R^{d_{\text{drug}}\times d}$ , and $P_{T}\in R^{d_{\text{drug}}\times d}$ are learnable parameter matrices. Next, the query matrix U , key matrix S , and value matrix T are uniformly divided into h heads according to the feature dimension, with each head having a dimension of $d_{h}=\frac{d}{h}$ . For each attention head i , the attention weight matrix $G^{i}$ and the corresponding fused feature $O^{i}$ of this head are calculated separately:

$$G^{i}=\text{softmax}\left(\frac{U^{i}{\left(S^{i}\right)}^{T}}{\sqrt{d_{h}}}\right)\in R^{n\times n},\;O^{i}=G^{i}T^{i}\in R^{n\times d_{h}}$$ (18)

The attention weight $G^{i}$ reflects the strength of the correlation between the circRNA sequence mode and the drug molecule graph mode across different samples, while $O^{i}$ is the weighted combination of the drug molecule graph mode features guided by correlation. Subsequently, the outputs of all attention heads are concatenated along the feature dimension, and integrated through a linear mapping matrix $P_{O}\in R^{d\times d}$ to obtain the final fused representation:

$$C=\text{Concat}\left(O^{0},O^{1},\ldots ,O^{h-1}\right)P_{O}\in R^{n\times d}$$ (19)

Through the multi-head attention mechanism, AFC can jointly capture the complex dependencies between the circRNA sequence mode and the drug molecule graph mode from multiple representation subspaces, thereby more comprehensively integrating cross-modal information. In the classification module, AFC employs a hierarchical MLP structure to conduct deep semantic encoding of the fused features, and combines BatchNorm technology to enhance the training stability and generalization ability of the model. Specifically, the fused features C are first input into a two-layer MLP:

$$L_{1}=\text{ReLU}\left(CA_{1}+c_{1}\right)\in R^{n\times d^{\prime }},\;L_{2}=\text{ReLU}\left(L_{1}A_{2}+c_{2}\right)\in R^{n\times d^{\prime \prime }}$$ (20)

Among them, $A_{1}\in R^{d\times d^{\prime }}$ and $A_{2}\in R^{d^{\prime }\times d^{\prime \prime }}$ are weight matrices, and $c_{1}\in R^{d^{\prime }}$ and $c_{2}\in R^{d^{\prime \prime }}$ are bias terms. To alleviate the problem of gradient vanishing or internal covariate shift in deep networks, we apply batch normalization after the activation output of each layer of the MLP:

$$L_{1}^{\prime }=\text{BatchNorm}\left(L_{1}\right),\;L_{2}^{\prime }=\text{BatchNorm}\left(L_{2}\right)$$ (21)

Ultimately, the normalized high-level features $L_{2}^{\prime }$ pass through a fully connected layer followed by a softmax activation function, resulting in the probability distributions corresponding to each class:

$$Y=\text{softmax}\left(L_{2}^{\prime }A_{3}+c_{3}\right)\in R^{n\times C}$$ (22)

Among them, $A_{3}\in R^{d^{\prime \prime }\times C}$ and $c_{3}\in R^{C}$ are the parameters of the classification layer, and C represents the number of categories. AFC combines the multi-head attention mechanism with the hierarchical regularization MLP structure, which not only enables dynamic and adaptive fusion of circRNA sequence modalities and drug molecule graph modalities, but also maintains good expression ability and generalization performance in complex tasks.

## 3 Experiment

### 3.1 Experimental setup and parameter settings

To ensure the reproducibility of our experimental results, we provide a comprehensive description of our implementation details and hyperparameter settings. All experiments were conducted under the same computational environment, and the following key parameters were utilized throughout the study: Network Architecture: The number of layers for both the GCN and GAT in our multi-view graph encoder was set to 3. The hidden dimension of this encoder was configured to 32. Training Strategy: The model was trained for 300 epochs. The Adam optimizer was employed with a learning rate of 0.001. Regularization: A Dropout rate of 0.3 was applied to mitigate overfitting. Reproducibility: A fixed random seed of 42 was used to guarantee the stability of all experimental outcomes and to facilitate replication. The rationale behind the selection of these hyperparameters, including the learning rate and model depth, is further supported by the sensitivity and ablation studies presented in subsequent sections.

### 3.2 Performance comparison

The five-fold cross-validation results, detailed in Table 1, demonstrate the remarkable stability of our model across different data partitions. The AUC values across all five folds exhibited minimal fluctuation, ranging narrowly from 0.8918 to 0.8962, yielding a high average AUC of 0.8940. This consistency, mirrored in other metrics such as AUPR, F1-score, Accuracy, and Recall, indicates that the model’s performance is not dependent on a particular split of the data and reliably reproduces the performance of the complete model. This low variance is a strong indicator of the model’s robustness. Other indicators, including AUPR, F1-score, Accuracy, and Recall, also showed similar consistency. This indicates that the model has good adaptability to different training-test data partitions. To further stress-test the model’s robustness, we conducted a more stringent ten-fold cross-validation. The results, presented in Table 2, reveal an even higher level of consistency across ten different data splits. The average AUC improved slightly to 0.8982, with values ranging from 0.8962 to 0.9001. This minor performance improvement under a more granular data division suggests that the model can effectively leverage larger training subsets. The consistently high performance and low standard deviations across all key metrics (AUPR, F1-score, etc.) in this setting provide compelling statistical evidence for the model’s generalizability and stability. The AUC values ranged from 0.8962 to 0.9001, with an average of 0.8982, and this result was even slightly higher than that of the five-fold cross-validation. Other key indicators such as AUPR (average 0.8973), F1-score (average 0.8354), Accuracy (average 0.8329), and Recall (average 0.8517) also remained at a high level, and the standard deviations between each fold were all within a relatively small range.

**TABLE 1:** The results of 5-fold cross-validation.

Metrics/Fold	AUC	AUPR	F1-score	Accuracy	Recall
1	0.8962	0.9028	0.8371	0.8332	0.8556
2	0.8923	0.8991	0.8330	0.8287	0.8502
3	0.8951	0.9015	0.8358	0.8319	0.8537
4	0.8918	0.8983	0.8319	0.8276	0.8489
5	0.8946	0.9013	0.8357	0.8311	0.8527
Average	0.8940	0.9006	0.8347	0.8305	0.8523

**TABLE 2:** The results of 10-fold cross-validation.

Fold	AUC	AUPR	F1-score	Accuracy	Recall
1	0.9001	0.8994	0.8385	0.8359	0.8551
2	0.8968	0.8953	0.8332	0.8302	0.8493
3	0.8974	0.8961	0.8341	0.8313	0.8504
4	0.8992	0.8986	0.8376	0.8352	0.8542
5	0.8962	0.8949	0.8324	0.8294	0.8486
6	0.8986	0.8979	0.8363	0.8339	0.8527
7	0.8978	0.8966	0.8350	0.8321	0.8515
8	0.8990	0.8982	0.8369	0.8346	0.8534
9	0.8965	0.8943	0.8321	0.8290	0.8481
10	0.8984	0.8970	0.8363	0.8333	0.8524
Average	0.8982	0.8973	0.8354	0.8329	0.8517

**FIGURE 2:**
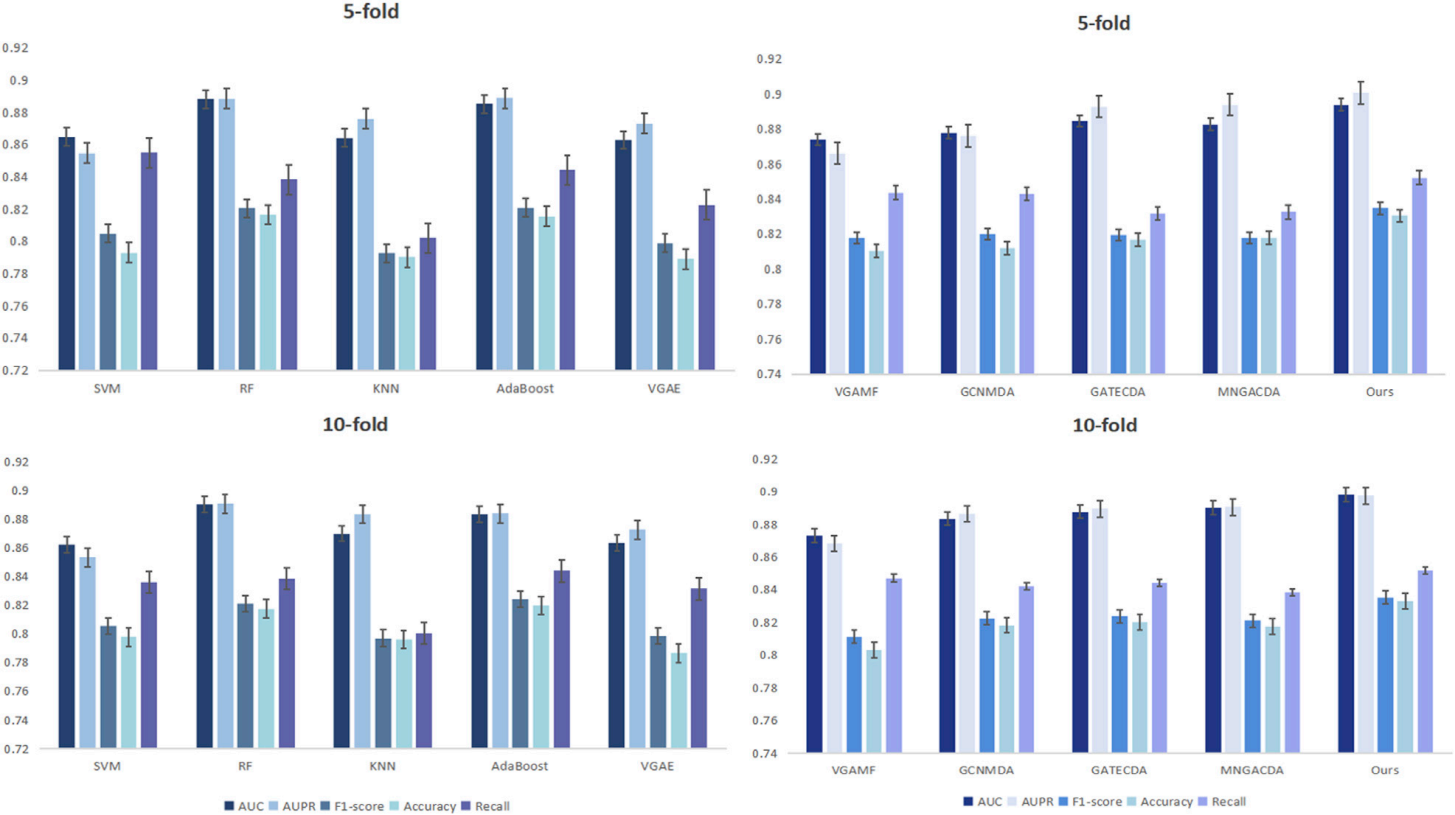
Performance comparison with the baseline under the 5-fold and 10-fold cross-validation.

Figure 2 can more intuitively show the comparison of the results of DMAGCL with the baseline model 5-fold and 10-fold cross-validation. The results of cross-validation fully demonstrate from a statistical perspective that the model has excellent generalization ability and stability. Under different data divisions, the model can maintain excellent performance, indicating that our method not only has a good fitting ability for the training data, but also has reliable predictive performance for unknown data.

We evaluated our dual-mask graph contrastive learning framework against several representative methods for circRNA-drug sensitivity association prediction. The baselines included traditional machine learning models (SVM, RF, KNN, AdaBoost) and recent graph neural networks (VGAE, VGAMF, GCNMDA, GATECDA, MNGACDA). All experiments were conducted under the same experimental environment and dataset, using five-fold and ten-fold cross-validation to ensure the statistical reliability of the results.

A comprehensive performance comparison against various baseline methods under five-fold cross-validation is summarized in Table 3. Our proposed method consistently outperforms all competitors, achieving the top scores across all five evaluation metrics. Notably, it attains an AUC of 0.8940 and an AUPR of 0.9006, representing a clear margin over the strongest baselines. The results reveal a key trend: graph neural network-based methods (e.g., GATECDA, MNGACDA) generally surpass traditional machine learning models (e.g., RF, SVM), underscoring the critical importance of explicitly modeling the graph structure for this prediction task. However, even among these advanced graph methods, our dual-mask framework establishes a new state-of-the-art. Including AUC, AUPR, F1-score, accuracy, and recall rate. The AUC reached 0.8940, and AUPR reached 0.9006, significantly outperforming other comparison methods. Traditional methods like RF and AdaBoost showed competitive results but were limited in capturing complex graph topology. Graph-based methods generally outperformed them, underscoring the importance of graph structure modeling. Notably, even attention-based methods like GATECDA and MNGACDA were outperformed by our framework, suggesting that our dual-mask strategy and contrastive learning paradigm better capture the intricate circRNA-drug associations than attention mechanisms alone.

**TABLE 3:** Performance comparison with the baseline under the 5-fold cross-validation.

Methods	AUC	AUPR	F1-score	Accuracy	Recall
SVM	0.8648	0.8547	0.8049	0.7928	0.8550
RF	0.8881	0.8885	0.8204	0.8165	0.8383
KNN	0.8642	0.8760	0.7926	0.7901	0.8020
AdaBoost	0.8852	0.8888	0.8207	0.8155	0.8443
VGAE	0.8628	0.8730	0.7988	0.7892	0.8227
VGAMF	0.8740	0.8662	0.8176	0.8104	0.8437
GCNMDA	0.8778	0.8762	0.8198	0.8119	0.8428
GATECDA	0.8846	0.8929	0.8194	0.8168	0.8316
MNGACDA	0.8826	0.8940	0.8178	0.8178	0.8326
Ours	0.8940	0.9006	0.8347	0.8305	0.8523

The superiority of our method is further confirmed under the more granular ten-fold cross-validation, as detailed in Table 4. Here, our model maintains its leading advantage with an average AUC of 0.8982 and AUPR of 0.8973. It is worth noting that while some baselines like GATECDA also showed improved performance with more folds, the performance gap between our method and these strong competitors persists. This consistent outperformance across both five-fold and ten-fold validations strongly suggests that the gains from our dual-mask contrastive learning framework are robust and not merely an artifact of a specific evaluation setup. Other indicators also show a similar trend. Compared with the five-fold cross-validation, the performance fluctuations of each method under the ten-fold validation are smaller, indicating that the method proposed in this paper has better adaptability to different data divisions. It is worth noting that in the ten-fold validation, the performance of the GATECDA method has improved compared to the five-fold validation, but its AUC (0.8876) and AUPR (0.8893) are still lower than that of the method proposed in this paper, thereby further demonstrating the superiority of the dual-mask contrastive learning framework we proposed in capturing complex biological association patterns.

**TABLE 4:** Performance comparison with the baseline under the 10-fold cross-validation.

Methods	AUC	AUPR	F1-score	Accuracy	Recall
SVM	0.8623	0.8533	0.8052	0.7977	0.8361
RF	0.8903	0.8906	0.8213	0.8176	0.8385
KNN	0.8700	0.8834	0.7970	0.7961	0.8005
AdaBoost	0.8836	0.8839	0.8240	0.8198	0.8439
VGAE	0.8634	0.8725	0.7987	0.7865	0.8314
VGAMF	0.8729	0.8682	0.8113	0.8030	0.8471
GCNMDA	0.8834	0.8864	0.8225	0.8183	0.8420
GATECDA	0.8876	0.8893	0.8237	0.8204	0.8442
MNGACDA	0.8901	0.8904	0.8210	0.8174	0.8383
Ours	0.8982	0.8973	0.8354	0.8329	0.8517

### 3.3 Ablation experiment

**FIGURE 3:**
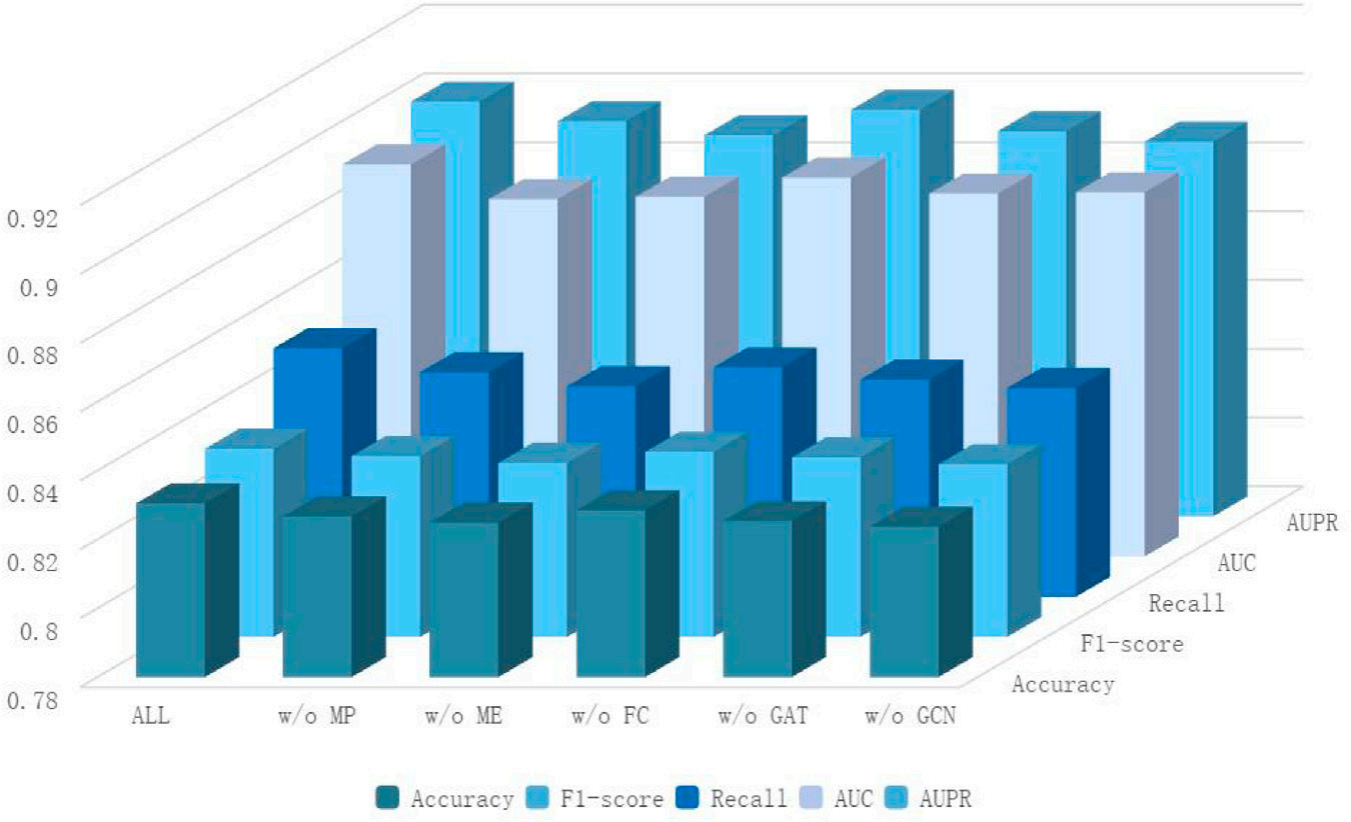
Ablation experiment results.

To dissect the contribution of each core component in our framework, we conducted a systematic ablation study, with results detailed in Table 5; Figure 3. We evaluated five ablated variants: removing the path mask (w/o MP), removing the edge mask (w/o ME), replacing the attention fusion classifier with an MLP (w/o FC), and removing either the GAT (w/o GAT) or GCN (w/o GCN) component from the encoder. The complete model achieves the best performance (AUC: 0.8940), confirming the synergistic design. The removal of either masking strategy (w/o MP or w/o ME) caused a substantial and nearly equivalent performance drop (AUC $\tilde{0}$ .884). This indicates that both path-level and edge-level masking are indispensable and complementary, each forcing the model to learn robust representations from different structural perspectives.

**TABLE 5:** Comparison of ablation experiment results.

Methods/Metrics	AUC	AUPR	F1-score	Accuracy	Recall
ALL	**0.8940**	**0.9006**	**0.8347**	**0.8305**	**0.8523**
W/o MP	0.8839	0.8949	0.8326	0.8267	0.8453
W/o ME	0.8846	0.8908	0.8306	0.8249	0.8412
W/o FC	0.8902	0.8982	0.8338	0.8285	0.8467
W/o GAT	0.8855	0.8920	0.8322	0.8253	0.8431
W/o GCN	0.8859	0.8889	0.8302	0.8237	0.8407

Bold values indicate the best performance in each metric.

The complete model achieved the best performance (AUC: 0.8940), demonstrating the synergy among its components. Removing either the path mask (w/o MP) or edge mask (w/o ME) caused a noticeable performance drop (AUC: 0.884). This indicates that both masking strategies contribute uniquely to learning robust representations: path masking helps capture higher-order dependencies, while edge masking forces the model to rely on broader neighborhood structures. When the path masking module was removed, it was observed that all indicators showed significant declines, especially the AUC value dropping to 0.8839 and the recall rate dropping to 0.8453. This phenomenon indicates that the path-level structural information plays an important role in capturing the complex association patterns between circRNAs and drugs. The path masking effectively enhances the model’s reasoning ability for graph structure dependencies by simulating the absence of local connectivity patterns. Similarly, the removal of the edge masking module also led to a significant performance reduction, with AUC dropping to 0.8846 and recall rate dropping to 0.8412. Edge masking weakens the direct connections between nodes at a finer granularity, prompting the model to pay more attention to other structural cues within the neighborhood. This strategy is crucial for learning robust graph representations. Notably, the performance decline of the path masking and edge masking modules was similar but with different focuses, indicating that the two masking strategies capture graph structure information at different levels, and their combined use can produce a complementary effect, jointly enhancing the model’s representation learning ability.

In terms of the feature fusion mechanism, when we replaced the attention-based feature fusion classifier with a simple multi-layer perceptron, the performance showed a slight but consistent decline, with AUC dropping to 0.8902. This result indicates that the attention mechanism can effectively allocate the importance weights of different features when integrating multi-view features, thereby improving the effect of feature fusion. However, the relatively small decline in performance also suggests that the MLP can still capture the interaction relationships between features to a certain extent, but lacks the ability to focus on important features prominently. The ablation experiments on the graph neural network components further revealed the complementary characteristics of GCN and GAT. When only using GCN and removing GAT, AUC dropped to 0.8855; while only using GAT and removing GCN, AUC dropped to 0.8859. GCN captures the local topological patterns of the graph through the aggregation of neighbor node features, while GAT dynamically emphasizes important structural features through attention weights. The combination of the two enables the model to fully utilize the regular structure of the graph and adaptively focus on key information.

### 3.4 Case experiment

In this study, representative anticancer drugs (Doxorubicin, Gefitinib, Sorafenib, and Paclitaxel) from the CTRP (Cancer Therapeutics Response Portal) database were selected as an independent validation set to test the model’s generalization capability and biological interpretability in real biological scenarios. Numerous existing works have utilized public drug response databases (such as CTRP, GDSC, CCLE) to validate the extrapolation performance of drug–gene or drug–RNA prediction models. Compared to these studies, the differences of this work lie in: (1) We predict circRNA–drug sensitivity associations, which represent a structured yet relatively sparse and noisy biological network problem; (2) The model employs multi-view encoding and various self-supervised enhancement strategies to improve the representation learning capability for complex graph structures, thereby aiming to maintain prediction accuracy even for unseen drugs/samples. By testing the model’s top 20 candidate circRNAs predicted on CTRP and comparing them with known experimental or literature-validated results in the database, we can directly compare our method with existing sequence/network-based or single GNN-based methods: if the validation rate on the independent database is significantly higher than random or baseline models, it indicates that our method holds an advantage in capturing biologically relevant signals, thereby establishing a connection with and surpassing relevant prior works. These drugs include doxorubicin, gefitinib, sorafenib, and paclitaxel, which represent different mechanisms of action of chemotherapy drugs and have good clinical representativeness and biological diversity. The experiment sorted the circRNA-drug sensitivity associations by prediction scores, selected the top 20 high-confidence associations in the CTRP database for independent external validation, and used the validation rate as the main evaluation indicator.

In the validation results of doxorubicin (Table 6), 15 out of the top 20 predicted circRNAs were validated, with a validation rate of 75%. It is noteworthy that the top 4 circRNAs, including HNRNPA2B1, CALD1, VIM, and COL3A1, were all validated as significantly associated, indicating that the model has a good ability to identify highly confident associations. Doxorubicin, as an anthracycline broad-spectrum anti-tumor drug, induces DNA damage by integrating into the DNA double helix and activates the apoptosis signaling pathway. These validated circRNAs may play an important regulatory role in its mechanism of action.

**TABLE 6:** The top 20 circRNA-drug sensitivity association prediction results of doxorubicin.

Ranking	circRNA	Verification	Ranking	circRNA	Verification
1	HNRNPA2B1	✓	11	COL4A2	✓
2	CALD1	✓	12	FBLN1	×
3	VIM	✓	13	LTBP3	✓
4	COL3A1	✓	14	KRT7	✓
5	LINC01089	×	15	CTTN	✓
6	DCN	×	16	COL6A2	✓
7	HMGA2	✓	17	TGFBI	✓
8	ANXA2	✓	18	PKM	×
9	FN1	✓	19	MGAT4B	✓
10	EFEMP1	✓	20	COL8A1	×

Gefitinib, as a selective EGFR tyrosine kinase inhibitor, is mainly used for the treatment of EGFR-mutated non-small cell lung cancer. According to the verification results (Table 7), 15 out of the top 20 predicted associations were verified, with a verification rate of 75%. It is worth noting that EFEMP2 ranked 10th was not verified, while HNRNPA2B1 ranked 18th was verified. This phenomenon indicates that the model still has room for optimization in the discrimination of certain moderate confidence associations, possibly related to the expression heterogeneity of circRNA in specific cell lines.

**TABLE 7:** The top 20 circRNA-drug sensitivity association prediction results of gefitinib.

Ranking	circRNA	Verification	Ranking	circRNA	Verification
1	VIM	✓	11	FBLN1	✓
2	CALD1	✓	12	LINC01089	×
3	COL6A2	✓	13	KRT7	✓
4	HSP90B1	✓	14	CTTN	✓
5	FN1	✓	15	TGFBI	✓
6	COL4A1	✓	16	DCN	×
7	ANXA2	✓	17	COL8A1	×
8	PKM	✓	18	HNRNPA2B1	✓
9	MGAT4B	✓	19	LTBP3	✓
10	EFEMP2	×	20	HMGA2	×

In the validation analysis of sorafenib (Table 8), the model demonstrated superior predictive performance, with 16 out of 20 predicted associations confirmed, yielding a validation rate of 80%. As a multi-target tyrosine kinase inhibitor, sorafenib is widely used in the treatment of hepatocellular carcinoma and renal cell carcinoma. Notably, all of the top six ranked circRNAs—including COL3A1, VIM, and ANXA2—were experimentally validated, indicating high prediction accuracy for top-ranking associations. These circRNAs may modulate tumor cell sensitivity to sorafenib through the regulation of multiple signaling pathways.

**TABLE 8:** The top 20 circRNA-drug sensitivity association prediction results of sorafenib.

Ranking	circRNA	Verification	Ranking	circRNA	Verification
1	COL3A1	✓	11	HSP90B1	✓
2	VIM	✓	12	FN1	✓
3	ANXA2	✓	13	KRT7	✓
4	CALD1	✓	14	LTBP3	✓
5	COL6A2	✓	15	CTTN	✓
6	FBLN1	✓	16	DCBLD2	✓
7	PKM	✓	17	HMGA2	×
8	LINC01089	×	18	MGAT4B	✓
9	HNRNPA2B1	✓	19	COL8A1	×
10	EFEMP1	✓	20	COL4A2	×

The most encouraging result comes from the validation analysis of paclitaxel (Table 9). Among the first 20 predicted correlations, 18 were validated, with a validation rate of up to 90%. Paclitaxel, as a microtubule stabilizer, inhibits microtubule depolymerization to block the cell cycle and is widely used in the treatment of breast cancer and ovarian cancer. This excellent predictive performance indicates that the dual-mask graph contrastive learning framework proposed in this paper has a special advantage for the prediction of sensitivity correlations of microtubule-targeted drugs. From the validation results, it can be seen that multiple cytoskeleton-related circRNAs including ANXA2, CALD1, and VIM have been validated, which is highly consistent with the mechanism of paclitaxel. Through the comprehensive analysis of the validation results of the four drugs (Table 10), the average validation rate of this model reached 80%, demonstrating good generalization ability. Further analysis revealed that some circRNAs were predicted to have high-ranking correlations in multiple drugs and were validated, such as VIM, CALD1, ANXA2, and FN1, etc. These circRNAs may be involved in the universal drug resistance mechanism of tumor cells, and their molecular functions are worthy of in-depth study through subsequent experiments. Additionally, the model’s predictions for some circRNAs (such as LINC01089, COL8A1) showed deviations, which may be related to the incomplete annotation or low expression level of these circRNAs in the CTRP database.

**TABLE 9:** The top 20 circRNA-drug sensitivity association prediction results of paclitaxel.

Ranking	circRNA	Verification	Ranking	circRNA	Verification
1	ANXA2	✓	11	FBLN1	✓
2	CALD1	✓	12	KRT7	✓
3	VIM	✓	13	COL6A2	✓
4	FN1	✓	14	PKM	✓
5	HSP90B1	✓	15	LINC01089	×
6	DCN	×	16	CTTN	✓
7	HMGA2	✓	17	LTBP3	✓
8	COL3A1	✓	18	MGAT4B	✓
9	EFEMP1	✓	19	TGFBI	✓
10	COL4A2	✓	20	HNRNPA2B1	✓

**TABLE 10:** Summary of the validation rates of the top 20 predicted associations for the four drugs.

Drug	Verified association count	Total number of associations	Verification rate
Doxorubicin	15	20	75%
Gefitinib	15	20	75%
Sorafenib	16	20	80%
Paclitaxel	18	20	90%
Average	16	20	80%

### 3.5 Parameter experiment

To determine the optimal parameter configuration of the model and to deeply understand the influence mechanism of each hyperparameter on the model’s performance, we conducted further parameter experiments on three key parameters: the number of GCN and GAT layers, the hidden layer dimension of the multi-view graph encoder, and the Dropout rate. These parameters respectively affect the model’s expression ability and generalization performance from three different dimensions: model depth, representation ability, and regularization strength. All experiments were conducted under the setting of five-fold cross-validation, with AUC and AUPR as the main evaluation indicators to ensure the statistical reliability of the results. Table 11 shows the impact of the number of GCN and GAT layers on the model’s performance. As the number of network layers increased from 1 layer to 3 layers, the model’s AUC improved from 0.8911 to 0.8940, and AUPR improved from 0.8957 to 0.9006. This indicates that appropriately increasing the network depth helps the model capture more complex graph structure features. However, when the number of layers continued to increase to 4 layers and 5 layers, the performance slightly decreased, with AUC dropping to 0.8914 and 0.8897 respectively. This might be due to the overfitting or gradient vanishing problem caused by an overly deep network. This phenomenon is consistent with the theoretical analysis of graph neural networks, that is, an excessively deep GNN layer may lead to the oversmoothing of node features, thereby reducing the model’s discriminative ability. Finally, 3 layers were selected as the optimal configuration for GCN and GAT, achieving a good balance between model expression ability and training stability.

**TABLE 11:** The impact of the number of GCN and GAT layers on performance.

GCN and GAT layers	AUC	AUPR
1	0.8911	0.8957
2	0.8922	0.8963
3	**0.8940**	**0.9006**
4	0.8914	0.8953
5	0.8897	0.8944

To determine the optimal learning rate for model training, we conducted a sensitivity analysis by evaluating a spectrum of values ranging from 0.1 to 0.00001. The performance, measured by AUC and AUPR, is summarized in Table 12. The results demonstrate that the learning rate significantly impacts model efficacy. A relatively high learning rate of 0.1 led to suboptimal performance (AUC = 0.8765, AUPR = 0.8691), suggesting potential instability during the gradient descent process. As the learning rate decreased to 0.01, the performance improved notably. The best performance was achieved with a learning rate of 0.001, yielding the highest AUC (0.8940) and AUPR (0.9006). Further decreasing the learning rate to 0.0001 and 0.00001 resulted in a slight but consistent performance degradation, indicating that overly small learning rates might hinder the model’s convergence to an optimal solution. Consequently, a learning rate of 0.001 was selected as the optimal configuration for all subsequent experiments, striking a balance between training efficiency and model performance.

**TABLE 12:** Comparison of model performance metrics.

Learning rate	AUC	AUPR
0.1	0.8765	0.8691
0.01	0.8864	0.8882
0.001	0.8940	0.9006
0.0001	0.8875	0.8927
0.00001	0.8868	0.8915

In terms of the selection of the hidden layer dimensions in the multi-view graph encoder, we compared different dimension settings ranging from 16 to 256. The experimental results are shown in Table 13. When the hidden layer dimension was set to 32, the model achieved the best performance, with an AUC of 0.8940 and an AUPR of 0.9006. Smaller dimension settings (such as 16) might limit the model’s representational ability, resulting in an AUC of only 0.8882; while overly large dimensions (such as 64, 128, 256) might introduce too many parameters, increasing the risk of overfitting and causing performance to decline to varying degrees. This result indicates that an appropriate hidden layer dimension can achieve the best balance between model capacity and generalization ability, ensuring sufficient feature representation space while avoiding optimization difficulties caused by parameter redundancy.

**TABLE 13:** The impact of hidden layers in multi-view graph encoders on performance.

Hidden layers	AUC	AUPR
16	0.8882	0.8943
32	**0.8940**	**0.9006**
64	0.8864	0.8938
128	0.8907	0.8983
256	0.8857	0.8924

Bold values indicate the best performance in each metric.

The Dropout rate, as a key parameter for controlling the regularization intensity of the model, has an impact as shown in Table 14. When the Dropout rate is set to 0.3, the model exhibits the best performance, with an AUC of 0.8940 and an AUPR of 0.9006. A lower Dropout rate (0.1) may lead to insufficient regularization, causing the model to overfit the training data; while a higher Dropout rate (0.5 and 0.7) may overly suppress the neuron activation, resulting in underfitting of the model, especially when the Dropout rate reaches 0.7, the performance significantly drops to an AUC of 0.8671 and an AUPR of 0.8783. This trend clearly demonstrates the dual role of the Dropout mechanism in deep learning models: an appropriate Dropout rate can effectively prevent overfitting and enhance the model’s generalization ability; but an overly strong Dropout rate will damage the model’s learning ability and lead to performance degradation.

**TABLE 14:** The impact of dropout rate on performance.

Dropout rate	AUC	AUPR
0.1	0.8907	0.8989
0.3	**0.8940**	**0.9006**
0.5	0.8859	0.8936
0.7	0.8671	0.8783

Bold values indicate the best performance in each metric.

Based on the results of the parameter experiments, the optimal parameter configuration of the model was determined: the number of layers for GCN and GAT is 3, the hidden layer dimension is 32, and the Dropout rate is 0.3. This configuration achieved the best performance in all three key parameters. Through this experiment, not only did it provide a reliable parameter basis for the method in this paper, but it also offered valuable references for other bioinformatics tasks based on graph neural networks.

## 4 Conclusion

This study introduces a dual-mask graph contrastive learning (DMAGCL) framework for predicting circRNA-drug sensitivity associations. The core innovation lies in its synergistic use of path- and edge-level masking to learn robust node representations, an adaptive contrastive loss to dynamically balance exploration and exploitation during training, and an attention fusion classifier to effectively integrate multi-modal features. Extensive evaluations confirmed that DMAGCL achieves state-of-the-art performance, with case studies on anti-cancer drugs demonstrating its biological relevance and an average verification rate of 80 Looking forward, this work opens several promising directions: First, the framework could be applied to related tasks like miRNA-disease association prediction, specifically to model the complex interactions in competing endogenous RNA (ceRNA) networks. Second, integrating multi-omics data (e.g., transcriptomics from specific cell lines) could help address the heterogeneity observed in some predictions and improve cell context-specific modeling. Finally, exploring the integration of large language models for biological sequence encoding could further enrich feature representations. This study thus establishes a robust and extensible computational paradigm with significant potential for biomarker discovery and drug sensitivity analysis in precision oncology.

## Data Availability

The data and code presented in the study are publicly available at https://github.com/yuqi23-guo/DMAGCL.
